# Derivation of age-adjusted LACE index thresholds in the prediction of mortality and frequent hospital readmissions in adults

**DOI:** 10.1007/s11739-020-02448-3

**Published:** 2020-07-28

**Authors:** Christopher Henry Fry, Erica Heppleston, David Fluck, Thang Sieu Han

**Affiliations:** 1 School of Physiology, Pharmacology and Neuroscience, University of Bristol, Bristol, BS8 1TD UK; 2 Quality Department, Ashford and St Peter’s NHS Foundation Trust, Guildford Road, Chertsey, Surrey KT16 0PZ UK; 3 Department of Cardiology, Ashford and St Peter’s NHS Foundation Trust, Guildford Road, Chertsey, Surrey KT16 0PZ UK; 4 Department of Endocrinology, Ashford and St Peter’s NHS Foundation Trust, Guildford Road, Chertsey, Surrey KT16 0PZ UK; 5 Institute of Cardiovascular Research, Royal Holloway, University of London, Egham, Surrey TW20 0EX UK

**Keywords:** Emergency medicine, Health economics, Public health, Likelihood ratio, ROC analysis

## Abstract

The LACE index has been shown to predict hospital readmissions and death with variable accuracy. A LACE index ≥ 10 is considered as high risk in the existing literature. We aimed to derive age-specific LACE index thresholds in the prediction of mortality and frequent readmissions. Analysis of prospectively collected data of consecutive alive-discharge episodes between 01/04/2017 and 31/03/2019 to a single hospital was conducted. The derivation of LACE index thresholds for predicting all-cause mortality within 6 months of hospital discharge or frequent readmissions (≥ 2 times within 28 days) was examined by receiver operating characteristics (ROC) in 32270 patients (14878 men, 17392 women) aged 18–107 year (mean = 64.0 years, SD = 20.5). For all patients with a LACE index ≥ 10, the area under the curve (AUC) for predicting mortality was 80.5% (95% CI 79.7–81.3) and for frequent readmissions was 84.0% (83.0–85.1). Two-graph ROC plots showed that the LACE index threshold where sensitivity equates specificity was 9.5 (95% intermediate range = 5.6–13.5) for predicting mortality and 10.3 (95% intermediate range = 6.6–13.6) for frequent readmissions. These thresholds were lowest among youngest individuals and rose progressively with age for mortality prediction: 18–49 years = 5.0, 50–59 years = 6.5, 60–69 years = 8.0, 70–79 years = 9.8 and ≥ 80 years = 11.6, and similarly for frequent readmissions: 18–49 years = 5.1, 50–59 years = 7.5, 60–69 years = 9.1, 70–79 years = 10.6 and ≥ 80 years = 12.0. Positive and negative likelihood ratios (LRs) ranged 1.5–3.3 and 0.4-0.6 for predicting mortality, and 2.5–4.4 and 0.3–0.6 for frequent readmissions, respectively, with stronger evidence in younger than in older individuals (LRs further from unity). In conclusion, the LACE index predicts mortality and frequent readmissions at lower thresholds and stronger in younger than in older individuals. Age should be taken into account when using the LACE index for identifying patients at high risk.

**Electronic supplementary material:**

The online version of this article (10.1007/s11739-020-02448-3) contains supplementary material, which is available to authorized users.

## Introduction

The number of emergency admissions to National Health Service (NHS) hospitals reached 6.6 million between December 2018 and November 2019 [1]. A recent report revealed that 13.8% of patients were readmitted within 30 days of hospital discharge during 2017–2018, rising from 12.5% in 2013–2014 [2]. Frequent readmissions are more costly than index admissions [3] and consume a large proportion of healthcare resources [4]. About 15–20% of readmissions are potentially avoidable [5] and reducing hospital readmissions has, therefore, been prioritised by healthcare services [6, 7]. Therefore, the ability to identify patients who are at risk of frequent readmissions would provide crucial information for effective healthcare service planning [8, 9]. The LACE index scoring algorithm provides such a tool [10], and has been implemented worldwide, including in the UK [11–13].

The LACE index score is based on Length of stay, Acuity of admission, Comorbidity and Emergency department visits, with a scale ranging from 0 to 19 [10]. The threshold at 10 of this index has been commonly used to indicate an increased likelihood of outcome risks, such as frequent readmissions and mortality [14–17]. However, the test accuracy of the LACE index for predicting readmissions and mortality has variably been reported [11–15]. Because advancing age is the ultimate determinant of mortality, the readmission frequency among older individuals diminish with years of follow up [18]. Thus, LACE index thresholds associated with a high risk of mortality and readmissions may vary with age. In this study, we examined the age-specific performance of the LACE index and derived LACE index thresholds to identify adults at increased risk of all-cause unplanned frequent readmissions and mortality after a hospital discharge.

## Methods

### Study design, participants and setting

We analysed prospectively collected data of consecutive alive-discharge episodes of unplanned admissions over two financial years between 1st April 2017 and 31st March 2019 to a single NHS. The data comprised clinical characteristics and care quality, including the length of stay and number of previous emergency department visits [19]. In line with the NHS data collection for general hospital admissions, cancer and obstetrics cases were excluded [2].

### Measurement

Morbidities were coded according to the international classification of diseases (ICD-10) for calculation of the Charlson co-morbidity index [20, 21]. Information on all-cause unplanned admissions and frequency of readmissions within 28 days, and all-cause mortality within 6 months and within 30 days of discharge from hospital was documented. The LACE index was computed from the length of stay (score range 0–7), acuity of admission (score 0 or 3), comorbidity (score range 0–5) and emergency department visits (score range 0 or 4) [22]—these scores summated to a scale of between 0 and 19 [10].

### Categorisation of variables

Frequent readmissions were defined as those who were readmitted two or more times within 28 days from an index discharge from hospital. Age was categorised by decades from 50 to 79 years: 50–59, 60–69, 70–79 and ≥ 80 years. All patients between 18 and 49 years were combined for the youngest age category due to lower mortality rates, and all those ≥ 80 years were also combined for the oldest category due to relatively small numbers of patients over 90 years.

### Statistical analysis

Receiver operating characteristic (ROC) curves were constructed to determine the area under the curve (AUC) for the LACE index as a predictor of outcomes (mortality or frequent admissions). Positive (LR+) and negative (LR−) likelihood ratios were calculated as $\frac{\text{sensitivity}}{{1{-}{\text{specificity}}}}$ and $\frac{{1 - {\text{sensitivity}}}}{\text{specificity}}$, respectively. Two-graph ROC curve analysis was conducted to optimise the selection of the maximum test accuracy for a given LACE index threshold value for identifying at-risk individuals, by plotting an overlapping graph of sensitivity and specificity curves as a function of the LACE index scores. The threshold *d*_0_ was obtained by interpolating from the intersection where sensitivity equals specificity (*θ*_0_), and the intermediate range (IR_95%_) was determined by the distance between the two points where sensitivity (lower limit) and specificity (upper limit) equal 95% [23–25]. ROC analysis was performed first for all patients and then for different age bands to obtain age-specific results. Analyses were performed using IBM SPSS Statistics, v23.0 (IBM Corp., Armonk, NY).

## Results

Data for a total of 32,270 patients (14,878 men) and (17,392 women) aged 18–107 years (mean = 64.0 years, SD = 20.5) were analysed. There were 29.3% of patients with a LACE index score of ≥ 10: 6.8% of patients died within 6 months (mean age of death = 81.2 years, SD = 12.1); 2.6% died within 30 days of hospital discharge (mean age of death = 81.5 years, SD = 12.0) and 3.3% of patients were readmitted ≥ 2 times within 28 days of hospital discharge. Among those aged 18–49 years (*n* = 8403), 50–59 years (*n* = 4304), 60–69 years (*n* = 4739), 70–79 years (*n* = 6068) and ≥ 80 years (*n* = 8756) respectively, the rates of mortality within six months of discharge were 0.5%, 2.0%, 4.8%, 7.7% and 15.6%, and within 30 days of discharge were 0.2%, 0.7%, 1.8%, 2.9% and 6.0%; frequent readmissions within 28 days of discharge were 1.0%, 1.5%, 2.4%, 3.3% and 6.7%. For clarity of presentation, only data for mortality within six months of discharge and readmissions within 28 days of discharge from hospital are presented subsequently. The results for mortality within 30 days of discharge, which closely follow the results of mortality within 6 months of discharge, are available in Supplementary material.

**Fig. 1:**
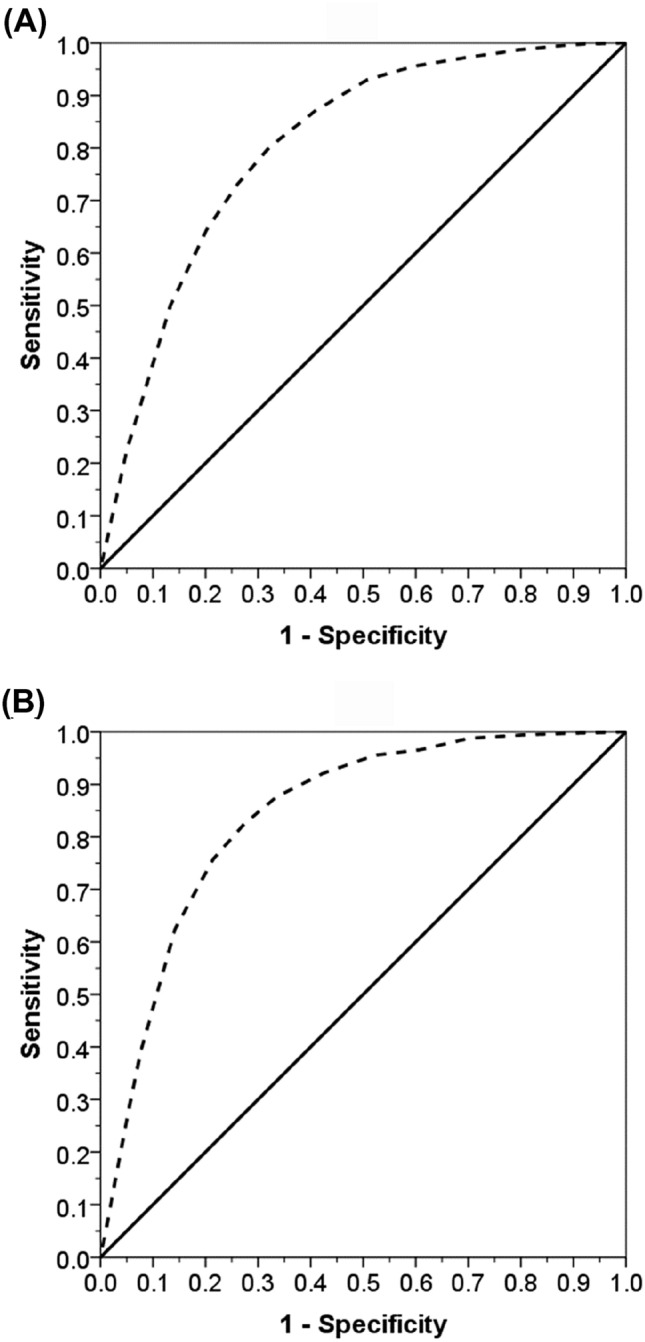
ROC curves (dotted lines) to estimate the ability of LACE to predict: **a** all-cause mortality within 6 months of discharge and **b** frequent admissions within 28 days of discharge from hospital, in adults aged 18–107 years. The solid 45° line (AUC = 50%) reflects a random classifier between the LACE index and either clinical outcome

ROC analysis to generate AUC values for all patients (18–107 years) showed that the LACE index as a predictor of mortality within 6 months of hospital discharge was 80.5% (95% CI 79.7–81.3) (Fig. 1a) and frequent readmissions were 84.0%, (95% CI 83.0–85.1) (Fig. 1b). Age-specific analysis of mortality showed that the AUC was highest among youngest individuals and diminished progressively with age: 18–49 years = 79.6%, 50–59 years = 78.1%, 60–69 years = 71.4%, 70–79 years = 72.0% and ≥ 80 years = 63.9% (Table 1). By contrast, analysis of frequent readmissions showed that AUCs (> 80%) were similarly high across different age groups, except for the oldest group where the value dropped to 76% (Table 1). Similar results were observed for the prediction of mortality within 30 days of hospital discharge (Supplementary Table 1).

**Table 1:** ROC analysis for all-cause mortality within 6 months or 30 days of discharge, and frequent readmission within 28 days of discharge from hospital based on a LACE index score ≥ 10

	*n*	Receiver operating characteristic analysis					
		Died within 6 months of hospital discharge			Readmitted ≥ 2 times within 28 days of hospital discharge		
		AUC (%)	95% CI	P*	AUC (%)	95% CI	P*
All patients (18–107 years)	32,270	80.5	79.7–81.3	< 0.001	84.0	83.0–85.1	< 0.001
Age bands							
18–49.9 years	8403	79.6	72.2–86.9	< 0.001	83.0	78.8–87.2	< 0.001
50–59.9 years	4304	78.1	73.3–82.9	< 0.001	85.5	81.9–89.1	< 0.001
60–69.9 years	4739	74.4	71.0–77.8	< 0.001	86.2	83.4–88.9	< 0.001
70–79.9 years	6068	72.0	69.6–74.4	< 0.001	83.7	81.6–85.8	< 0.001
≥ 80 years	8756	63.9	62.3–65.4	< 0.001	76.0	74.4–77.6	< 0.001

*P value significantly different form AUC = 50%

Table 2 shows that within each of the five age categories (18–49 years, 50–59 years, 60–69 years, 70–79 years and 80 years), the LR+ values were 3.34, 2.53, 2.09, 1.96 and 1.53, and LR− values were 0.40, 0.39, 0.41, 0.45 and 0.63 for age-specific LACE thresholds for predicting the probability of mortality within 6 months of hospital discharge. The corresponding LR+ values were 3.30, 3.60, 4.38, 3.32 and 2.51, and LR− values were 0.42, 0.28, 0.32, 0.26 and 0.59 for predicting frequent readmissions within 28 days of discharge from hospital.

**Fig. 2:**
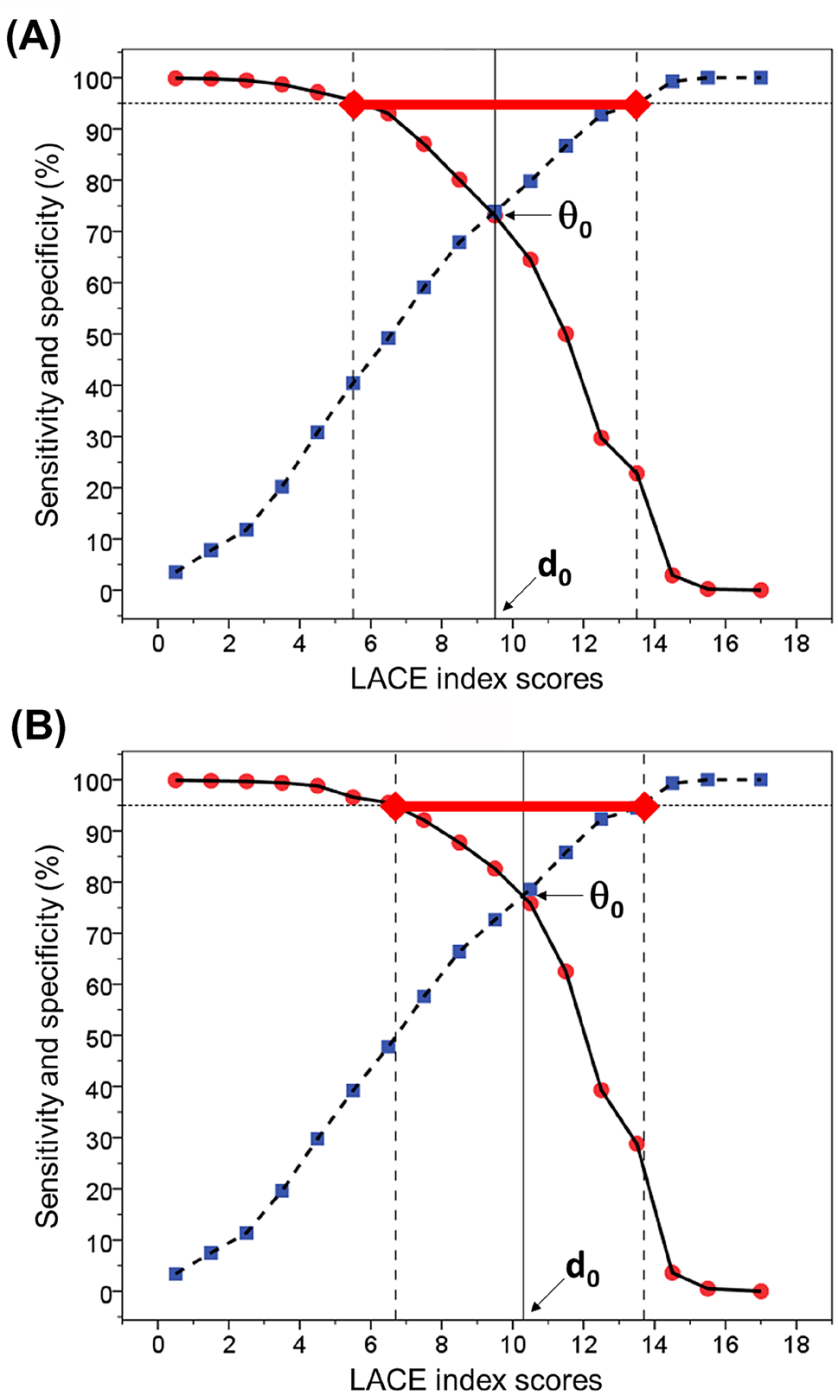
Two-graph ROC plot to identify: **a** mortality within 6 months and **b** frequent admissions within 28 days of discharge from hospital. These show the threshold of the LACE index (*d*_0_) interpolated from the point where sensitivity (filled circle) equals specificity (filled square) (*θ*_0_), and the intermediate range (red bar) where sensitivity = 95% (lower limit) and specificity = 95% (upper limit) in adults aged 18–107 years

For all patients, two-graph ROC plots showed the LACE index threshold where sensitivity equals specificity for predicting mortality within 6 months was 9.5 (IR_95%_ = 5.6–13.5) (Fig. 2a) and frequent readmissions were 10.3 (IR_95%_ = 6.6–13.6) (Fig. 2b). The LACE index threshold for predicting mortality within 6 months was lowest among youngest individuals and rose progressively with age: 18–49 years = 5.0, 50–59 years = 6.5, 60–69 years = 8.0, 70–79 years = 9.8 and ≥ 80 years = 11.6, and similarly for frequent readmissions: 18–49 years = 5.1, 50–59 years = 7.5, 60–69 years = 9.1, 70–79 years = 10.6 and ≥ 80 years = 12.0 (Table 3). Results from analysis of the LACE index in relation to mortality and frequent readmissions within 30 days of hospital discharge showed similar patterns (Supplementary Table 1).

**Table 2:** Likelihood ratios for age-specific LACE thresholds to estimate the probability of mortality within 6 months of discharge and frequent readmissions within 28 days of discharge from hospital

Age bands	Died within 6 months of hospital discharge		Readmitted ≥ 2 times within 28 days of hospital discharge	
	LR+	LR−	LR+	LR−
18–49 years	3.34	0.40	3.30	0.42
50–59 years	2.53	0.39	3.60	0.28
60–69 years	2.09	0.41	4.38	0.32
70–79 years	1.96	0.45	3.32	0.26
≥ 80 years	1.53	0.63	2.51	0.59

*LR+* positive likelihood ratio; *LR−* negative likelihood ratio

**Table 3:** LACE index thresholds where sensitivity equals specificity (*θ*_0_) and 95% intermediate range derived from two-graph ROC plots

	*n*	LACE index threshold (*θ*_0_) and 95% intermediate range (IR)			
		Died within 6 months of hospital discharge		Readmitted ≥ 2 times within 28 days of hospital discharge	
		*θ*_0_	95% IR	*θ*_0_	95% IR
All patients (18–107 years)	32,270	9.5	5.6–13.5	10.3	6.6–13.6
Age bands					
18–49.9 years	8403	5.0	0.8–7.8	5.1	3.0–7.7
50–59.9 years	4304	6.5	3.7–10.0	7.5	5.1–9.8
60–69.9 years	4739	8.0	3.9–11.8	9.1	6.7–11.8
70–79.9 years	6068	9.8	5.6–13.1	10.6	8.1–13.2
≥ 80 years	8756	11.6	7.2–14.4	12.0	10.3–14.4

Among the 44 patients in the youngest age group (18–49 years) who died within 6 months of hospital discharge, only eight patients (18.2%) were identified using LACE index threshold at 10 compared with 35 patients (79.5%) who were identified using the age-specific threshold at 5. However, the false-positive rate rose from 1.6% to 33.2% when the LACE threshold was lowered from 10 to 5. Similarly, among 87 patients in the youngest age group with frequent readmissions within 28 days of hospital discharge, only 15 (17.2%) individuals were identified using LACE index threshold at 10 compared with 75 (86.2%) individuals who were identified using LACE index threshold at 5. The use of age-specific LACE index thresholds continues to identify more patients at risk of death or frequent readmissions up to the age of 69 years. There were fewer oldest patients (≥ 80 years) being identified by an age-specific LACE index threshold of 11.5 (Supplementary Table 2). The patterns were similar for the relationship between age-specific LACE index thresholds and mortality with 30 days of hospital discharge.

## Discussion

This study over a period of 2 years found that a high LACE index (≥ 10) was associated significantly to all-cause mortality within 6 months of discharge and frequent admissions within 28 days of discharge from hospital. The thresholds of LACE index values where sensitivity equals specificity (maximum test accuracy) for identifying mortality and frequent admissions were lowest among younger individuals and rose progressively with age. As far as we are aware, this is the first study to demonstrate an age-specific association of the LACE index with mortality and frequent readmissions.

The results observed in this study show the test accuracies of the LACE index to predict mortality or frequent readmissions are relatively high compared (> 80%) with those in the published literature. Previous studies have reported a test accuracy of the LACE index to be between 55% and 82% to predict readmissions [11–15], and between 66.3% and 83% to predict mortality after a hospital discharge [11, 13, 15]. These differences may be due to varying underlying conditions of the patients or their age range. We, therefore, extended our analysis further to assess an age-specific performance of the LACE index. It showed that the test accuracy was highest among the youngest age group and declined progressively with age in its ability to predict mortality. Moreover, test accuracy to predict frequent readmissions was generally high (AUCs > 83%) up to 79 years, after which AUC dropped to 76% for the oldest group (≥ 80 years). The poorer performance by the LACE index to predict readmissions among older individuals may be due to higher mortality rates among this age group. Although health insurance status in countries, such as the US [26], where private health insurance is required, has been shown to influence readmission rates, this factor has no impact on the UK population since all aspects of emergency care are covered by the NHS.

Using two-graph ROC analysis, we have demonstrated that the thresholds of the LACE index to identify mortality and frequent admissions vary widely with age. Thus, the use of a single LACE index threshold at 10 to define high risk is not applicable for all ages, particularly for the less-studied younger individuals. It is important to emphasise that the two-graph ROC analysis (Fig. 2) is employed to facilitate objective decisions on desired LACE index thresholds. When a desired LACE index threshold is selected, a number of factors should then be taken into account, including clinical benefits and risks, costs of interventions and physiological characteristics (such as age) of the individual [27]. Since the value for *θ*_0_ was below a preselected accuracy level (95%), two cut-off values representing the upper and lower limits of IR_95%_ were considered as the borderline range for the clinical interpretation of test results [23]—if high sensitivity is desired, the LACE index threshold is lowered towards the lower limit of IR_95%_ but specificity is compromised. Conversely, raising the LACE index threshold towards the upper limit of IR_95%_ would reverse these conclusions. The IR can also be varied, e.g. by lowering the points where sensitivity and specificity equal 90% (IR_90%_), which would result in a narrower IR [28].

Likelihood ratios are useful statistics for measuring the accuracy of a diagnostic test in clinical practice [29]. They have a number of advantages over sensitivity and specificity or predictive values: they can capture the size of abnormality of test results, are independent of disease prevalence, therefore, are more stable than predictive values when prevalences vary, and they can be used at the individual patient level [30]. Report of likelihood ratios is, therefore, recommended for inclusion in clinical studies. The likelihood ratio is derived from the ratio of the probability of a given test result in patients with poor outcome (i.e. mortality or frequent readmissions) to the probability of the same test result in patients without poor outcome. The further likelihood ratios are from unity, the stronger the evidence for the presence or absence of adverse outcome: LR+ over 10 and LR− less than 0.1 indicate strong evidence to confirm or exclude the diagnosis of interest, respectively [31]. We observed that the likelihood ratios for predicting mortality (LR+ range: 1.5–3.3 and LR− range: 0.4–0.6) and frequent readmissions after hospital discharge (LR+ range: 2.5–4.4 and LR− range: 0.3–0.6) were relatively modest, but generally the evidence was stronger in younger than in older individuals.

Findings from this study indicate that interventional plans to reduce frequent readmissions should be directed at younger individuals who have the greatest probability of future readmissions. There is a misconception that to reduce readmissions, it is necessary to focus targeted interventions at “high-risk” groups, such as the very ill or very old (over 80 years), who actually account for a relatively small share of a total number of admissions (27% in this study). It has been argued that because the majority of admissions are from low-risk individuals, significant risk reduction can only be achieved if interventions to reduce risk factors were applied to the whole population [32]. An effort to reduce readmissions of older people, while neglecting younger individuals, may also suffer from the regression to the mean effect [33]. This phenomenon was well demonstrated in a study by Roland et al. [18] It was observed that patients ≥ 65 years with ≥ 2 admissions were responsible for 38% of admissions in the index years. However, without any intervention this reduced to about 10% in the following years and further to 3% of admissions 5 years later. This is likely to be due to high mortality rates among older individuals—data in this study showed that up nearly 20% of individuals over 70 years who were admitted ≥ 2 times within 28 days of hospital discharge died within 6 months compared with < 5% among those younger than 60 years. Thus, by lowering the “high-risk” LACE index score to our derived threshold values of as low as 5 for younger individuals would encompass the majority of those who account for most admissions in the long term. We found that less than 20% of the youngest group who died within 6 months of hospital discharge were identified by a LACE index threshold of > 10, whilst up to 80% were identified when the threshold was lowered to 5. More patients were also identified by age-specific thresholds up to 69 years. These findings were further reinforced by similar results from age-specific LACE index thresholds in the prediction of frequent readmissions.

If the LACE index threshold was raised from 10 to 11.5 for the oldest group, then the total numbers would be smaller but have a greater proportional mortality rate, i.e. there would be fewer false negatives while missing some with a score of 10 who will die, but not be picked up by this stratification. On the other hand, by staying at 10, more false positives are introduced and some true positives will be missed. Thus, the balance between resources and optimising recognition of risk should be taken into account when selecting a threshold. We found that those with a LACE score ≥ 10, 1127 deaths were identified. When the threshold was increased to 11.5, there were 867 deaths with a score above this threshold (a reduction of 23.1%).

The strengths of this study lie in its large number of patients which enable us to derive age-specific thresholds of LACE index in the identification of mortality in a wide range of age (18 to 107 years). We define frequent readmissions for those who were admitted ≥ 2 times within 28 days. Certain limitations may arise from patients who have left our catchment area, particularly younger age groups who may find new jobs or have other social commitments—this would inevitably alter readmission rates. Although the accuracy of the test is a fair parameter for describing the diagnostic performance of the test itself, it doesn’t necessarily influence clinical decision-making. The validation of pre-specified sensitivity and specificity of LACE index thresholds could be more useful if a clinical strategy could be followed after the application of the test to establish whether reductions in mortality and frequent readmissions could be achieved.

In conclusion, the LACE index predicts mortality and frequent readmissions with greater accuracy and at lower thresholds in younger compared to older individuals. Age should be taken into account when using the LACE index for identifying patients at high risk. However, clinical usefulness of this index still depends on the validation of specific thresholds and on the definition of a specific strategy for mortality and frequent hospital readmissions.

## Electronic supplementary material

Below is the link to the electronic supplementary material.

Supplementary material 1 (DOCX 30 kb)

## Abbreviations

AUC: Area under the curve
ICD: International classification of diseases
IR: Intermediate range
LACE: Length of stay, acuity of admission, comorbidity and emergency department visits
LR: Likelihood ratio
NHS: National Health Service
ROC: Receiver operating characteristic
