# Supplementary material for: Derivation of age-adjusted LACE index thresholds in the prediction of mortality and frequent hospital readmissions in adults
Source: Intern Emerg Med. 2020 Jul 28;15(7):1319–25. doi: 10.1007/s11739-020-02448-3 (PMC7511461; doi:10.1007/s11739-020-02448-3)
Supplement: Supplementary file 1 — Supplementary material 1 (DOCX 30 kb) [file 11739_2020_2448_MOESM1_ESM.docx]

**Supplementary** **Table 1.** LACE index thresholds where sensitivity equals specificity (θ_0_) and 95% intermediate range derived from two-graph ROC plots.

|  | Receiver operating characteristic analysis for patients who died within 30 days of hospital discharge | | | LACE index threshold (θ_0_) and 95% intermediate range (IR) | |
| --- | --- | --- | --- | --- | --- |
|  | AUC (%) | 95% CI | *p* | θ_0_ | 95% IR |
| All patients (18-107 years) | 81.4 | 80.2-82.7 | <0.001 | 9.8 | 6.3-13.7 |
| Age bands |  |  |  |  |  |
| 18-49.9 years | 81.6 | 68.4-94.7 | <0.001 | 6.5 | 1.0-7.8 |
| 50-59.9 years | 78.8 | 69.9-87.6 | <0.001 | 6.5 | 1.8-9.5 |
| 60-69.9 years | 71.6 | 66.3-76.9 | <0.001 | 8.5 | 5.6-11.9 |
| 70-79.9 years | 73.0 | 69.4-76.6 | <0.001 | 10.0 | 5.8-13.2 |
| ≥80 years | 67.5 | 65.3-69.7 | 0.011 | 11.6 | 7.2-14.4 |

**P*-value significantly different form AUC = 50%.

**Supplementary** **Table 2.** Age-specific mortality rates at different LACE cut-offs levels.

|  | LACE index | Mortality (*n*) | LACE index | Mortality (*n*) | χ^2^ | *p* | LACE index | Mortality (*n*) | LACE index | Mortality (*n*) | χ^2^ | *p* |
| --- | --- | --- | --- | --- | --- | --- | --- | --- | --- | --- | --- | --- |
| Died within six months of discharge | | | | | | | | | | | | |
| 18-49.9yr | <10 | 36 | ≥10 | 8 | 71.2 | **<0.001** | <5.0 | 9 | ≥5.0 | **35** | **42.2** | **<0.001** |
| 50-59.9yr | <10 | 70 | ≥10 | 17 | 33.1 | **<0.001** | <6.5 | 24 | ≥6.5 | 63 | 78.5 | **<0.001** |
| 60-69.9yr | <10 | 108 | ≥10 | 118 | 187.8 | **<0.001** | <8.0 | 60 | ≥8.0 | 166 | 137.7 | **<0.001** |
| 70-79.9yr | <10 | 134 | ≥10 | 332 | 219.7 | **<0.001** | <10.0 | 134 | ≥10.0 | 332 | 219.7 | **<0.001** |
| ≥80yr | <10 | 242 | ≥10 | 1127 | 180.0 | **<0.001** | <11.5 | 502 | ≥11.5 | 867 | 225.8 | **<0.001** |
| Died within 30 days of discharge | | | | | | | | | | | | |
| 18-49.9yr | <10 | 12 | ≥10 | 3 | 29.8 | **<0.001** | <5.0 | 2 | ≥5.0 | **13** | **19.1** | **<0.001** |
| 50-59.9yr | <10 | 21 | ≥10 | 8 | 27.0 | **<0.001** | <6.5 | 8 | ≥6.5 | 21 | 25.8 | **<0.001** |
| 60-69.9yr | <10 | 47 | ≥10 | 37 | 39.2 | **<0.001** | <8.0 | 28 | ≥8.0 | 56 | 32.4 | **<0.001** |
| 70-79.9yr | <10 | 46 | ≥10 | 131 | 93.6 | **<0.001** | <10.0 | 46 | ≥10.0 | 131 | 93.6 | **<0.001** |
| ≥80yr | <10 | 70 | ≥10 | 459 | 102.8 | **<0.001** | <11.5 | 155 | ≥11.5 | 374 | 153.0 | **<0.001** |
| Frequent readmissions within 28 days of discharge | | | | | | | | | | | | |
| 18-49.9yr | <10 | 72 | ≥10 | 15 | 125.8 | **<0.001** | <5.0 | 12 | ≥5.0 | **75** | **109.8** | **<0.001** |
| 50-59.9yr | <10 | 42 | ≥10 | 23 | 112.0 | **<0.001** | <6.5 | 8 | ≥6.5 | 57 | 107.3 | **<0.001** |
| 60-69.9yr | <10 | 30 | ≥10 | 82 | 236.5 | **<0.001** | <8.0 | 11 | ≥8.0 | 101 | 139.5 | **<0.001** |
| 70-79.9yr | <10 | 20 | ≥10 | 183 | 230.3 | **<0.001** | <10.0 | 20 | ≥10.0 | 183 | 230.3 | **<0.001** |
| ≥80yr | <10 | 20 | ≥10 | 569 | 255.3 | **<0.001** | <11.5 | 83 | ≥11.5 | 506 | 432.0 | **<0.001** |
